# Effect of Grinding and Regenerative Heat Treatment on the Fracture Resistance of a Zirconia/Porcelain Veneer Interface

**DOI:** 10.3290/j.jad.b2701695

**Published:** 2022-03-01

**Authors:** Lucas Miguel Candido, Eduardo Bellini Ferreira, Lígia Antunes Pereira Pinelli

**Affiliations:** a Dentist, Department of Dental Materials and Prosthodontics, São Paulo State University (Unesp), School of Dentistry, Araraquara, São Paulo, Brazil. Idea, hypothesis, experimental design and execution, wrote the manuscript.; b Assistant Professor, Department of Materials Engineering, São Carlos Engineering School, University of São Paulo (USP), São Carlos, São Paulo, Brazil. Idea, hypothesis, experimental design and execution, wrote the manuscript.; c Associate Professor, Department of Dental Materials and Prosthodontics, São Paulo State University (Unesp), School of Dentistry, Araraquara, São Paulo, Brazil. Idea, hypothesis, experimental design and execution, wrote the manuscript.

**Keywords:** zirconia, dental prosthesis, x-ray diffraction, surface properties, heat treatment

## Abstract

**Purpose:**

To experimentally assess the effect of regenerative heat treatment (HT) on yttria-stabilized tetragonal zirconia polycrystalline ceramic (Y-TZP) to guarantee veneer adhesion strength.

**Materials and Methods:**

One surface of bar-shaped Y-TZP specimens was ground (G) with a diamond stone, while the control samples (C) were not. Groups C900 and G900 were submitted to HT at 900°C for 60 min, whereas groups C1000 and G1000 were submitted to HT at 1000°C for 30 min. The treated surfaces were characterized by x-ray diffractometry (XRD), scanning electron microscopy (SEM), and optical and mechanical profilometry. The energy release rate through interface fracture was determined by a four-point bending test on notched Y-TZP veneered specimens. XRD was refined by the Rietveld method, mean roughness (Ra) and energy release rate were submitted to two-way ANOVA (α = 0.05), and the images were analyzed descriptively.

**Results:**

The monoclinic phase (vol%), means of Ra (µm), and the energy release rate (J/m^2^) were, respectively: C = 1.2/0.17/6.8, C900 = 0.0/0.18/6.6, C1000 = 0.0/0.18/7.6, G = 2.6/1.16/8.3, G900 = 0.0/1.07/8.0, and G1000 = 0.0/1.01/5.7. The surface fraction of monoclinic zirconia increased by grinding and decreased by HT. Ra also increased after grinding (p < 0.005) but remained unaltered after HT (p = 0.22). Increased irregularity was observed in the G groups and a subtle smoothing of the surface after HT. After the fracture of the bilayers, a residual amount of porcelain could be seen on the zirconia surface in all groups. The energy release rate was statistically equal among all groups (p > 0.05).

**Conclusion:**

Heat treatment after grinding completely restored the tetragonal phase of zirconia without altering the energy release rate during interfacial fracture.

Zirconia-based biomaterials have been extensively studied due to their promising properties.^12,34,47^ At atmospheric pressure, zirconia (ZrO_2_) presents three polymorphic phases: monoclinic (m), tetragonal (t), and cubic (c); all of them depend on temperature and mechanical factors and are directly related to the properties of the materials.^2,16,34,47^ The expansion (~4.5 vol%) provoked by the t→m transformation can weaken the microstructure due to microcracking during cooling after the sintering process, which makes the ceramic useless. This phase transformation can be managed through doping with yttria (Y_2_O_3_), since – if crack propagation occurs – yttria causes the expansion to close the fissure, consequently hindering the fracture and making the material tougher. These yttria-stabilized tetragonal zirconia polycrystalline ceramics (Y-TZP) have been used in dentistry since the 1990s because of their beneficial features, such as high strength and fracture resistance, biocompatibility, interesting optical properties, high hardness, wear resistance, high acid and alkali corrosion resistance, and elastic modulus similar to steel.^11,12,16,34,45,47^ Given such advantages, Y-TZP is used for the fabrication of metal-free prostheses, implants, abutments, and orthodontic brackets.^12,47^Additionally, it is particularly suited to stand the high stresses on multiple-tooth prostheses in posterior regions.^42^

In Y-TZP, neighboring grains constrain the tetragonal phase from expanding and transforming into the monoclinic phase. However, the strain energy released by crack propagation relieves the microstructure constraints that keep the tetragonal phase metastable, allowing it to change into monoclinic grains, thus expanding and compressing the crack tip, which in turn prevents it from further propagation.^10,12,34,47^ At the same time, the increased diameter of transformed zirconia grains may exceed the critical size for microcracking, and a crack propagating in the new microstructure would branch and lose energy, which constitutes a second toughening mechanism.^10^

The stresses during mechanical grinding of Y-TZP can induce t→m transformation.^19,25,33^ Some authors point out that the grinding procedure used to adjust a Y-TZP infrastructure may compromise the future interface with a porcelain veneer due to the presence of monoclinic grains.^13,20,23-25^ Besides reducing toughness, the monoclinic grains in the material can accelerate long-term degradation processes in aqueous or acidic environments, reducing the composite strength.^10,24,25^

Grinding is often performed by prosthodontists and dentists to adjust the prosthesis infrastructure both internally and externally, in an attempt to improve adhesion of the porcelain veneer.^8,25,31,38^ Adhesion failure leads to delamination.^2,3,40^

Delamination can occur through different mechanisms, such as thermal expansion coefficient incompatibility between the infrastructure and the veneer, surface defects or improper infrastructure support, overload during use, fatigue, and low fracture resistance.^3,6,41^ As a result, the purpose of grinding is to modify the zirconia surface by increasing roughness or surface energy, resulting in mechanical improvement of interlocking and wettability, which consequently favors the adhesive interface^1,15,37^ despite possibly damaging^8,13,20,23,25,26,30^ the zirconia surface.^1,28,30,37^ Some authors suggest heat treatment (HT) after grinding and before veneering with porcelain as a way to reduce the damage of grinding by welding shut any microcracks, thus relieving residual stresses and reversing the t→m transformation on the surface.^7,15,21,26,35,36,38,44^ In these terms, heat treatment would be regenerative.^15^

Despite satisfactory results,^7,35,36,38,44^ there is no consensus regarding the HT protocol. Furthermore, there are no studies on the effect of HT on the surface and mechanical properties of a dental ceramic bilayer based on Y-TZP regarding the adhesion strength between the two ceramic layers.

Many studies have tried to improve the adhesion between ceramic layers by using different methods, such as zirconia surface blasting, application of liners with different compositions, grinding, laser, ultrasound, or modification of the cooling step after the porcelain veneering process.^1,4,27,29,30,32,46^ Some authors reported the effect of heat treatment on the zirconia/porcelain interface.^15,29^ However, they used this technique on polished zirconia or after blasting. The literature contains no study on adhesion tests on surfaces subjected to heat treatment after grinding.

To evaluate the adhesion between zirconia and a porcelain veneer, most authors perform a shear-bond strength test, which often shows a cohesive failure of the porcelain veneer.^15,48^ Nevertheless, this test may not give the real value of the interfacial strength,^49^ since non-uniform stresses during testing may mask the results. Charalambides et al^9^ proposed an analytical method to calculate the fracture effect on the interface. This test has been used with dental materials,^14,43,50^ but only a few studies were carried out with zirconia.^17,49^ Further investigation of new, easier methods to increase the longevity of zirconia/porcelain prostheses is of great importance for prosthodontist and dentists. Therefore, the present work aims to evaluate the effect of grinding and heat treatment on the surface microstructure and roughness of Y-TZP as well as the bond strength of zirconia/porcelain veneers. The null hypothesis was that HT would not change the Y-TZP microstructure or its bond strength to a porcelain veneer.

## Materials and Methods

Blocks of a partially sintered Y-TZP (Lava Frame, 3M Oral Care; St Paul, MN, USA) were cut into bars of 25 x 5 x 1.9 mm (group G, ground) and 25 x 5 x 1.5 mm (group C, unground control) in a metallographic cutting machine (Isomet 1000, Buehler; Lake Bluff, IL, USA) using a diamond saw (Series 15LC Diamond, Buehler) and water irrigation. The groups consisted of 45 specimens each. The initial dimensions were designed considering 20% linear shrinkage after the final sintering and 0.3 mm of substance loss in the G group. The nomenclature for the experimental groups is shown in Table 1.

**Table 1:** Nomenclature of the tested groups

Group	Grinding	Heat treatment
C	No	No
C900	No	900°C / 60 min
C1000	No	1000°C / 30 min
G	Yes	No
G900	Yes	900°C / 60 min
G1000	Yes	1000°C / 30 min

All groups consisted of sintered Y-TZP.

Silicone tips (Exa-cerapol, Edenta Dental; Au, Switzerland) were used to remove irregularities after cutting. The veneer surfaces were then sequentially polished with 1200-, 1500-, and 2000-grit silicon-carbide sandpapers (401Q, 3M Oral Care), and the specimen dimensions were measured with a digital caliper (500-144B, Mitutoyo; Suzano, SP, Brazil). Specimens were sintered according to the manufacturer’s protocol in a Lava 200 furnace (Dekema Dental-Keramiköfen; Freilassing, Germany), resulting in specimens with dimensions of 20 x 4 x 1.5 mm for group G and 20 x 4 x 1.2 mm for group C. With the aid of an automatic grinding device described elsewhere,7 specimens of group G were ground (0.3 mm) without water irrigation with a medium-grit cylindrical diamond stone (MasterCeram, 133-104-SDN, Polierwerk; Straubenhardt, Germany) attached to a handpiece (LB 100 Electric Prosthetic Bench Micromotor, Beltec; Araraquara, SP, Brazil) at a speed of approximately 20,000 rpm.21

Groups C900, C1000, G900, and G1000 were heat treated in a laboratory ceramic oven (AluminiPress, EDG Equipamentos e Controles; São Carlos, SP, Brazil) at 900°C for 60 min (groups C900 and G900) and 1000°C for 30 min (groups C1000 and G1000), based on a previous study.^7^ The specimens were placed in the oven preheated to the treatment temperature and treated isothermally. After heat treating, the samples were removed from the oven and cooled down to room temperature on the bench. X-ray diffractometry (n = 2) was performed to evaluate the reversibility of the t→m phase transformation using a Bruker D8 Advance diffractometer (Bruker; Karlsruhe, Germany) with copper anode l K1 = 1.5406 Å and Ka_2_ = 1.5444 Å and a IKa_2_:IKa_1_ intensity ratio of 0.5, between 20 and 80 degrees with a step size of 0.02 degrees and a step time of 3 s in continuous scan mode.

The phases m and t were identified with the crystalline structures described by Gualtieri et al^18^ and Bondars et al.^5^ After phase identification, the results were refined by the Rietveld method for quantitative analysis.^39^

The arithmetic mean roughness (Ra) was measured (n = 10) in a Mitutoyo SJ 400 profilometer (Mitutoyo; Yokohama, Japan) with a reading accuracy of 0.01 μm, reading length of 2.5 mm, operating tip speed of 0.5 mm/s, and effective tip radius of 5 μm. Three measurements were performed on each specimen, and the average was calculated. The measurement spots were the same for all groups: one in the center and two equidistant (5 mm) from the center in the longitudinal direction of the specimen. The measurements were performed in the opposite direction of the grinding lines on the groups submitted to this procedure.

The topographic analysis was carried out using a PB1000 optical profilometer (n = 2) (Nanovea; Irvine, CA, USA) over an area of 500 x 500 µm with a 5-µm pitch. The image was processed with Gwyddion 2.48 software (GPL free software, Department of Nanometrology, Czech Metrology Institute; Okružní, Beroun, Czech Republic). For surface characterization, two additional specimens from each group were cleaned in an ultrasonic cleaner with acetone (5 min), distilled water (5 min), and isopropyl alcohol (5 min), then coated with carbon and analyzed using scanning electron microscopy (SEM) in an Inspect F50 microscope (FEI; Eindhoven, Netherlands). SEM was also used to analyze the crack path (n = 2) and the fracture surface (n = 2) after the same cleaning and metallization protocols mentioned above.

Low-fusion nano-fluorapatite porcelain (IPS e.max Ceram, Ivoclar Vivadent; Schaan, Liechtenstein) was applied to the conditioned surface of the Y-TZP bars to compose bilayer bodies 2.4 mm in height.^17^ In group G, the porcelain was built up in several layers applied to the ground Y-TZP surface. First, the bars were cleaned in an ultrasonic cleaner with water and dried with absorbent paper. The porcelain was built up in layers of different materials, deposited from powder suspensions with a #3 marten-hair brush (Kolinsky, Kota; Cotia, SP, Brazil): 1) liner (IPS e.max Zirliner + IPS Zirliner Build-Up Liquid allround); 2) wash (IPS e.max Ceram Dentin A4 + IPS Build-UP Liquid allround); 3) two layers of dentin (IPS and e.max Ceram Dentin A4 + IPS Build-UP Liquid allround); and 4) glaze (Glaze paste, Ivoclar Vivadent). After applying each layer, the specimens were fired in a Programat P310 oven (Ivoclar Vivadent), following the protocol recommended by the manufacturer (Table 2).

**Table 2:** Firing schedule of the IPS e.max Ceram system

Material	iT (°C)	DT (min)	q (°C/min)	FT (°C)	HT (min)	V1 (°C)	V2 (°C)	OP (°C)
Zirliner	403	4	40	960	1	450	959	–
Wash	403	4	40	750	1	450	749	–
Dentin	403	4	40	750	1	450	749	–
Glaze	403	6	60	725	1	450	724	450

iT: initial temperature; DT: drying time; q: heating rate; FT: final temperature; HT: holding time; V1: vacuum start temperature; V2: vacuum final temperature; OP: oven opening temperature.

The liner was approximately 0.1 mm thick. It was followed by a layer of conventional porcelain (wash). A dentin layer was then molded with the aid of a hard silicone form (Zetalabor, Zhermack; Rovigo, Italy). The second firing (second dentin layer) was performed without the form to correct imperfections; the second dentin layer was applied in excess on the top surface and sides for later adjustment and smoothing. The specimens were corrected with a fine-grit grinding wheel Gr. 140/170 (Dinser Diamond Tools; São Paulo, SP, Brazil) to make plane and parallel surfaces. The sample side surfaces were further corrected with handpiece in which were mounted a diamond cutter (Edenta) and rubber tips (126c, Edenta). Finally, a thin, uniform glaze layer was applied. After porcelain veneer buildup, the dimensions of the final specimens were 20.0 x 4.0 x 2.4 mm (length x width x height) with a Y-TZP/porcelain thickness ratio of 1:1.^9,18^

A 0.7-mm-deep notch was made in the center of the veneer parallel to the short axis of the sample, so that a fracture could initiate from a crack with known length. The notch was formed using a 7020 KG double-sided diamond wheel (KG Sorensen; São Paulo, SP, Brazil), with the final depth limited by a dial indicator coupled to the grinding device. The depths were verified using a Leica optical microscope (Leica Microsystems; Wetzlar, Germany) with the Leica Application Suite EZ software.

The energy release rate (G) was used to evaluate the adhesion between the porcelain veneer and zirconia. Four-point bending tests were performed on the bilayer specimens (n = 13) in a mechanical testing machine (EMIC DL2000, Testing Equipment and Systems; São José dos Pinhais, PR, Brazil) with a 5-kN load cell and actuator speed of 0.1 mm/min.^49^

The equation proposed by Charalambides et al^9^ was used to calculate the energy release rate as follows:

$$G=\frac{\eta (P^{2}l^{2}(1-\upsilon_{1}^{2}))}{E_{1}b^{2}h^{3}}$$

where *P* is the load at the steady region observed in the curve of load vs displacement due to interface crack propagation, *l* is the distance between the inner and outer rollers on the same side, *E*_1_ and ν_1_ are Young’s modulus and Poisson ratio of zirconia (200 GPa and 0.32), respectively,^17^ and *b* and *h* are the width and total thickness of the specimen, respectively. The non-dimensional parameter η was calculated by:

$$\eta =\left[\frac{1}{{\left(h_{1}|h\right)}^{3}}-\frac{\lambda }{{\left(h_{2}|h\right)}^{3}+\lambda {\left(h_{1}|h\right)}^{3}+3\lambda (h_{1}h_{2}|h^{2})/(h_{2}|h+\lambda (h_{1}|h))}\right]$$

h = , where *h*_1_ and *h*_2_ are the thickness of zirconia and porcelain layers, respectively. λ was calculated as follows:

$$\lambda =\frac{(1-\upsilon_{2}^{2})\;E_{1}}{(1-\upsilon_{1}^{2})\;E_{2}}$$

where E_1_ and ν_1_ are Young’s modulus (70 GPa) and Poisson ratio and the Poisson ratio (0.27) of the porcelain, respectively.^49^

The XRD results were refined by the Rietveld method. SEM and optical profilometry were analyzed descriptively. The values of *Ra, P,* and *G* were checked by the Shapiro-Wilk test (α = 0.05), and subsequently analyzed by two-way ANOVA followed by the Tukey’s post-hoc (α = 0.05) test. The BioEstat 5.0 software (Belém, Pará, Brazil) was used for statistical calculations.

## Results

**Fig 1:**
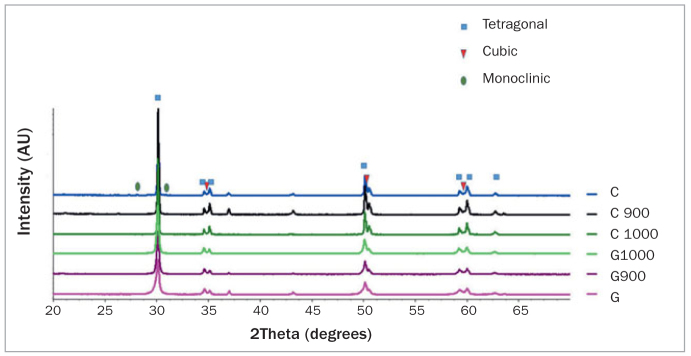
Phase diffractograms of the experimental groups.

Figure 1 shows the results of the XRD analyses for the different experimental groups and the expected diffraction peak positions of the corresponding crystalline phases.

Table 3 shows the volume fraction of monoclinic, tetragonal, and cubic zirconia determined by the Rietveld analysis. As can be seen, grinding increased the number of monoclinic and cubic phases on the specimen surface. The heat treatments reversed the t→m transformation in both groups, extinguishing the monoclinic phase and bringing the tetragonal volume fraction approximately back to the control level.

**Table 3:** Volume fraction (vol%) of the Y-TZP monoclinic (m), tetragonal (t) and cubic (c) phases, n = 2

Heat treatment	Grinding					
	without			with		
	m	t	c	m	t	c
No	1.2	85.9	12.9	2.6	66.1	31.3
900°C/60 min	0.0	82.3	17.7	0.0	85.8	14.2
1000°C/30 min	0.0	82.5	17.5	0.0	88.5	11.5

From Table 4, it is apparent that grinding increased the arithmetic mean roughness, Ra (p < 0.005), although heat treatment did not cause any change either in the control (C) or the ground (G) group (p = 0.22). There was no interaction between groups and treatments (p = 0.15).

**Table 4:** The arithmetic mean roughness, Ra (µm), and corresponding standard deviations of the Y-TZP sample groups subjected to different grinding and heat treatments (n = 10)

Heat treatment	Grinding	
	without	with
No	0.17 ± 0.04^Aa^	1.16 ± 0.24^Ab^
900°C/60 min	0.18 ± 0.03^Aa^	1.07 ± 0.08^Ab^
1000°C/30 min	0.18 ± 0.03^Aa^	1.01 ± 0.11^Ab^

Different superscript lowercase letters indicate a statistical difference between columns. Different superscript uppercase letters indicate a statistical difference between rows.

**Fig 2:**
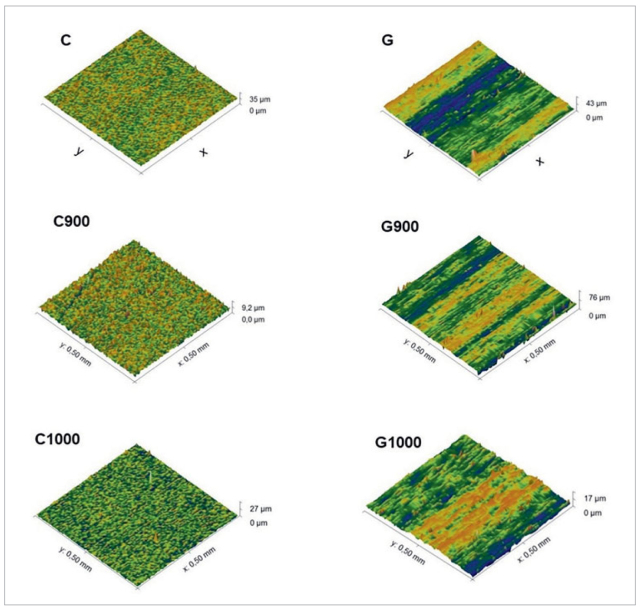
Optical profilometry of the experimental groups.

Optical profilometry (Fig 2) shows the increase of surface roughness in group G. No difference was observed between the different heat treatments.

**Fig 3:**
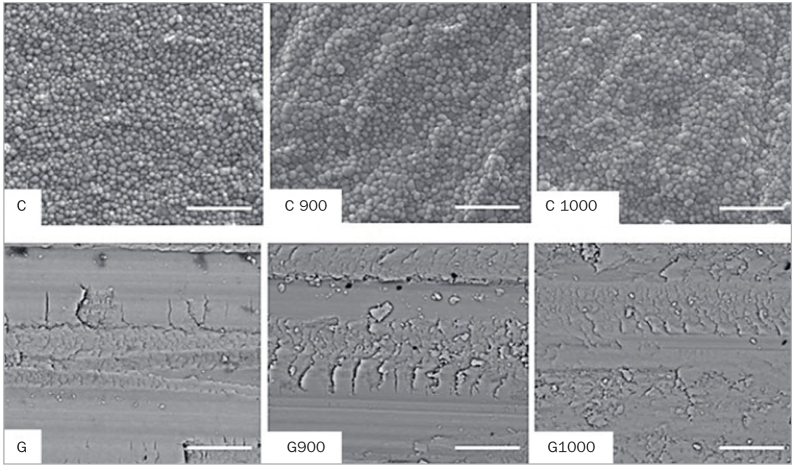
Superficial topography of the experimental groups. The scale bar corresponds to 5 µm (magnification approximately 8000X).

The SEM images (Fig 3) exhibit a zirconia surface with a regular microstructure and well-preserved grains, contours and shapes in non-ground groups. Grinding was also responsible for increasing the irregularity in the surface microstructure, producing longitudinal grooves parallel to the grinding direction, deformations, scale-like structures, debris and cracks. In turn, HT produced only subtle changes, causing the chips to be a little less evident and with rounded corners.

**Fig 4:**
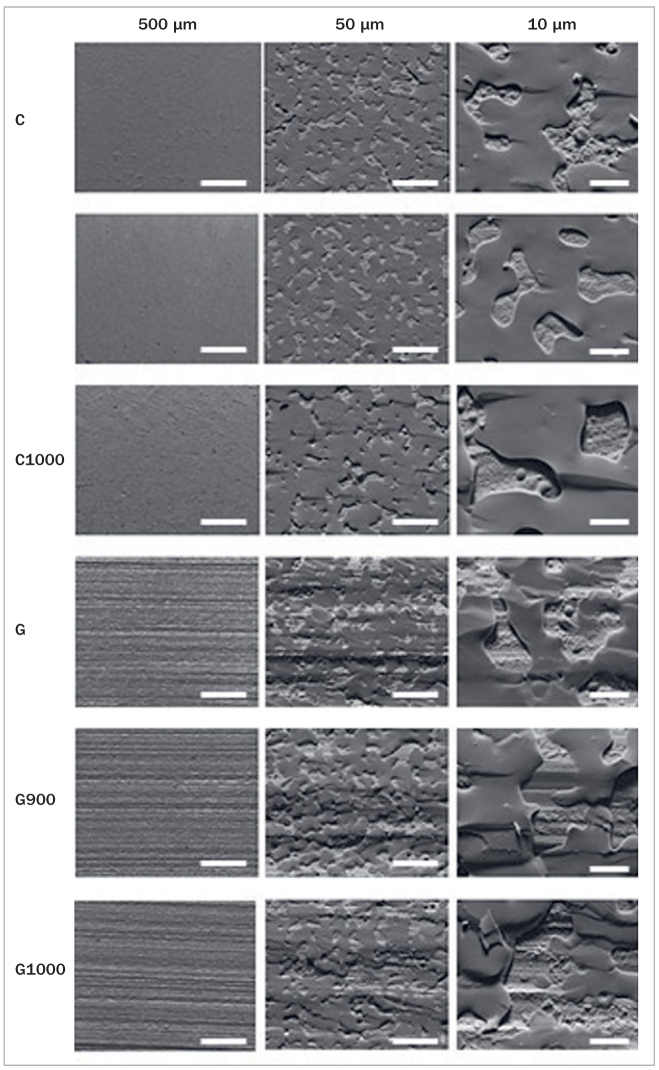
Superficial topography of the experimental groups after the four-point bending test. The magnifications are approximately 140X, 1400X, and 5000X from the first to third column, respectively.

**Fig 5:**
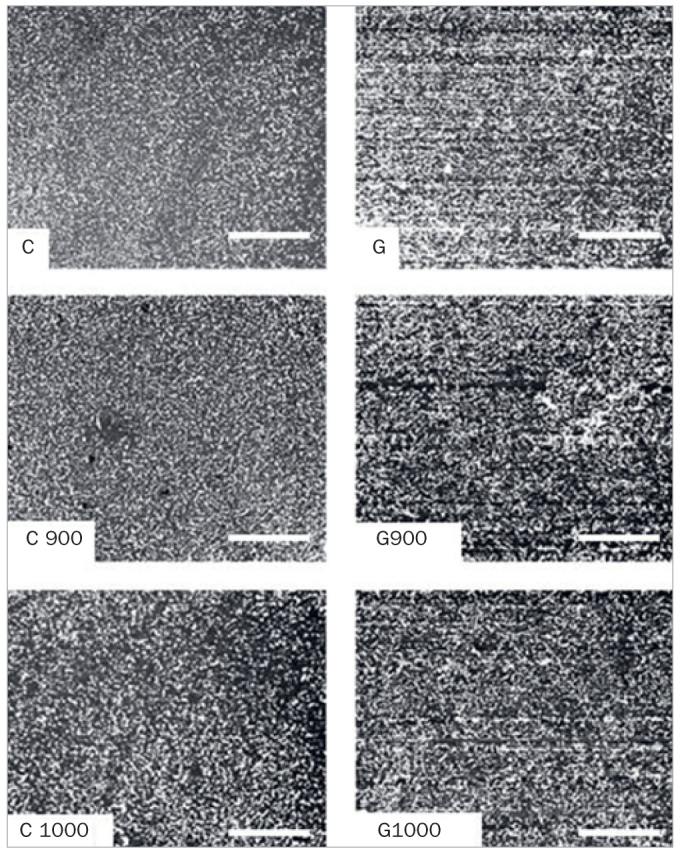
Superficial topography of the experimental groups after the four-point bending test (backscattered electron mode). The light phase is zirconia, and the dark phase is porcelain (magnification 140X).

The SEM fracture surface analysis (Figs 4 and 5) showed that part of the porcelain veneer remained attached to the zirconia surface, probably the first layer (liner), in a similar manner in all groups. The phase contrast in backscattered electron images (Fig 5) shows that all groups were similar.

**Fig 6:**
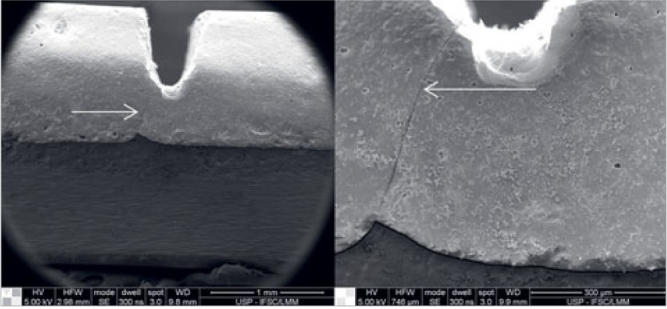
Image of a crack (arrow) at the end of the notch. Confirmation for the adhesion strength test (magnifications 30X and 120X).

Figure 6 shows that a crack started next to the notch end and spread through the interface, where it is possible to see zirconia detachment in some regions and liner remains in others. Thus, the fracture can be classified as mixed, cohesive, and adhesive.

**Fig 7:**
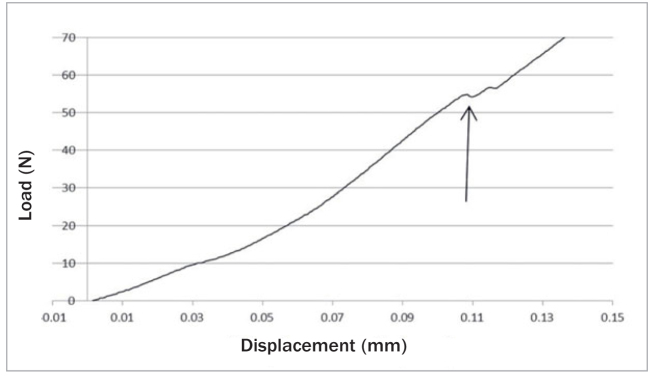
Graph plotted from the four-point bending test. The arrow points to the load in the region of steady crack propagation (*P*).

The *P* load in the region of steady crack propagation (Table 5) was determined from the plots of the load as a function of dislocation (Fig 7) and was used to calculate the strain energy release rate *G* in the interface fracture (Table 6). For* P,* although interaction between groups and treatments (p = 0.02) was apparent, no statistical difference was found between groups with and without grinding (p = 0.42) or between the heat treatments (p = 0.15). The values of *G* followed the same behavior of *P*, ie, there was an interaction between groups and treatments (p = 0.005) but no statistical difference was found between grinding or not grinding (p = 0.52), nor between the heat treatments (p = 0.29).

**Table 5:** The average *P* load (N) in steady crack propagation and corresponding standard deviation for different experimental conditions (n = 13)

Heat treatment	Grinding	
	with	without
No	51.4 ± 10.9	55.8 ± 3.8
900°C/60 min	48.6 ± 8.7	55.0 ± 7.3
1000°C/30 min	52.2 ± 7.5	46.0 ± 8.5

There was no statistical difference between groups.

**Table 6:** Strain energy release rate *G* (J/m^2^), average and standard deviation of the different experimental groups (n = 13)

Heat treatment	Grinding	
	without	with
No	6.8 ± 2.1	8.3 ± 2.0
900°C/60 min	6.6 ± 1.4	8.0 ± 2.7
1000°C/30 min	7.6 ± 1.3	5.7 ± 1.7

There was no statistical difference between groups.

## Discussion

Zirconia-based prostheses are increasingly common, and adjustments by grinding are still necessary, even using CAD/CAM technology.^8,21^ It is essential to know the kind of damage these adjustments can cause to the material and how to reverse it without impairing the adhesion of the porcelain veneer in order to increase the longevity of such prostheses. The present study aimed to evaluate the surface microstructure and roughness of Y-TZP after grinding and heat treatment as well as their effect on the bond strength with a porcelain veneer. The null hypothesis regarding lack of significant differences between the factors analyzed as a function of heat treatments was partially accepted, since there was an influence on the evaluated properties.

Grinding can influence the Y-TZP phase transformation,_8,13,20,23,25,26,38_ and factors such as abrasive grain size, generated heat, grinding time, exerted pressure and cutting tool efficiency are associated with the tetragonal to monoclinic phase transformation (t→m).^20,24,30,38^ XRD analysis (Fig 1 and Table 3) showed that grinding increased the number of monoclinic phases on the surface, which is in accordance with the literature.^1,20,23,25,30,38^ The t→m transformation can produce a toughening mechanism, in which the tetragonal phase transforms into larger monoclinic grains due to crack propagation, consequently compressing the crack tip.^1,23,33^ This transformation may increase the material strength,^30,38,49^ but decrease its ability to prevent further cracking,^30,38^ promote long-term degradation and weaken the adhesion of the porcelain veneer.^15,23,33,40^

An increase in the cubic phase was also observed during the grinding procedure. This may be a rhombohedral (*r*) phase from partially transformed tetragonal zirconia. Denry and Kelly also identified this phase after grinding.^13^ According to those authors, the *r* phase can be classified as a deformed *t *phase, the formation of which is closely associated with stress.

Heat treatments at 900°C for 60 min or 1000°C for 30 min can reverse the t→m transformation, which is congruent with the behavior already reported in the literature.^7,21,35,36^ In addition, the *r *phase was also stabilized in the *tt* phase as observed by Denry and Kelly,^13^ maintaining the material with its crystallographic conformation similar to that observed before grinding. Zirconia with fewer *m* and *r* phases shows less long-term degradation^10,13,47^ and maintains the number of *t* phases and its ability to prevent microcracks, making it more resistant.^12,13,34,47^

Grinding also increases Y-TZP roughness (Table 4), as observed in the literature.^7,20,33,38,44^ However, none of the studies that evaluated HT after grinding concomitantly assessed its effect on surface roughness. In the present study, for both the C and G groups, HT did not influence the surface roughness. In group G alone, a small decrease in *Ra* from 1.16 to 1.01 µm after HT was observed, yet with no statistical significance. These changes were corroborated by optical profilometry (Fig 2) and SEM (Fig 3). In addition, the zirconia surface in the C group was regular, with preserved grain boundaries and shapes. Surface grinding increased the irregularity of the specimen’s microstructure in this region. Longitudinal grooves were observed in the direction of grinding, together with deformations, scale-like structures, debris, and superficial cracks, as reported in the literature.^7,19,21,33,36^ Unlike the group C, where no change was perceived after HT, in group G, the treatment only promoted small changes, causing the chips to be a little less evident with rounding of the sharp corners, which is in accordance with the literature.^15,21,35,36^

The method proposed by Charalambides et al^9^ was used to assess the adhesive strength by the strain energy release rate (*G*) due to crack propagation in the interface. This method is believed to be more precise, since it can evaluate the interface between materials accurately.^9,14,17,43,49,50^ Neither grinding nor HT interfered with the strain energy release rate (Table 6), ie, they did not influence the adhesion between the zirconia and the porcelain veneer. Therefore, grinding was not sufficient to improve adhesion, despite observations to the contrary by Qeblawi et al.^37^ In a literature review, Lundberg et al^30^ were also unable to verify the efficacy of grinding in improving adhesion and also pointed out that sandblasting is even better, which illustrates how controversial the subject is. Regarding HT, no statistically significant difference was observed. The modifications as a result of HT, as observed by SEM, were not enough to improve adhesion. 

The values of *G* in the present study were similar to those obtained by Wang et al^49^ and Göstemeyer et al,^17^ who also studied the zirconia/porcelain interface. Such values are lower than those for metal-ceramics.^43^ Suansuwan et al^43^ obtained values close to 39.4 J/m^2^ for nickel-chrome infrastructures. Yamada et al^50^ found values of approximately 80 J/m^2^ after a gold-based treatment to increase the interfacial energy in titanium/porcelain bodies. These values are higher than those obtained for zirconia, which increases the chance of delamination in prostheses with a Y-TZP infrastructure. The high values for metal-ceramic prostheses can be explained by the chemical bond between the porcelain and the metallic core, which is not observed in zirconia infrastructures.^48^ Several studies and methods have been performed to improve the zirconia/porcelain interface^28,30^ with satisfactory results with respect to blasting^30^ and laser^32^ on the zirconia surface. More recently, a sonochemical method was tested by Bastos et al,^4^ but it did not increase adhesion.

Corroborating the strain energy release results, the micrographs of the fractured interface surface (Figs 4 and 5) show that a large amount of porcelain, probably mostly liner, adhered to the zirconia. In general, from Fig 5 demonstrates the similarity between all groups. Despite some limitations, eg, bar-shaped specimens have geometry different from that of teeth, this is not an in vivo study, and that we did not consider cyclical loads as those observed in chewing, the post-grinding heat treatment can be performed under both tested conditions (1000°C for 30 min and 900°C for 60 min) without affecting the adhesion of the porcelain veneer. Studies with crown-shaped specimens and clinical studies still have to be performed to precisely assess the properties examined here.

## Conclusion

In this study, grinding increased the volume fraction of monoclinic zirconia and surface roughness as well as Y-TZP superficial scratches, microcracks, and deformation. Heat treatment reversed the t→m transformation and had a slight influence on the visual aspect of the surface (smoothed the edges, mainly after the 1000°C/30min treatment), but did not alter the surface roughness. The strain energy release rate during fracture of the zirconia/porcelain veneer interface was not affected by grinding or heat treatment.

## Clinical relevance:

After the adjustments of zirconia by grinding, heat treatment can reverse the transformation and potentially restore the protective toughening of the restoration without affecting the strain energy release rate during fracture of the zirconia/porcelain veneer interface.
